# Quantifying Inter- and Intramolecular Interactions in Liquids with Correlated Vibrational Spectroscopy: Case Study of CCl_4_ and CH_3_CN

**DOI:** 10.1021/acs.jpcb.5c03893

**Published:** 2026-01-12

**Authors:** M. Flór, V. Vorobev, A. Bouchez, A. Marchioro, D. M. Wilkins, S. Roke

**Affiliations:** 1 Laboratory for Fundamental BioPhotonics (LBP), Institute of Bioengineering (IBI), School of Engineering (STI), 27218 École Polytechnique Fédérale de Lausanne (EPFL), Lausanne CH-1015, Switzerland; 2 Centre for Quantum Materials and Technologies, School of Mathematics and Physics, 1596 Queen’s University Belfast, Belfast, Northern Ireland BT7 1NN, United Kingdom; 3 Institute of Materials Science and Engineering (IMX), School of Engineering (STI), École Polytechnique Fédérale de Lausanne (EPFL), Lausanne CH-1015, Switzerland; 4 Lausanne Centre for Ultrafast Science, École Polytechnique Fédérale de Lausanne (EPFL), Lausanne CH-1015, Switzerland

## Abstract

Correlated vibrational spectroscopy (CVS) is a hyper-Raman-based vibrational spectroscopy that retrieves separate spectra of individual (self-correlated, SC) and interacting (cross-correlated, CC) molecules. The spectra are recorded in the >40 cm^–1^ THz/mid-IR frequency range and contain modes that are IR and/or Raman active. Here, we further develop CVS and apply it to investigate intra- and intermolecular interactions using room temperature liquid carbon tetrachloride (CCl_4_), a nonpolar liquid, and acetonitrile (CH_3_CN), a polar liquid, as case studies. CVS spectra of CCl_4_ display no intermolecular coupling, confirming the isotropy and short-range nature of the molecular interactions. Strong intramolecular coupling is observed on the Fermi resonance, and the relative phase between the participating modes is determined based on the intensities in the experimental spectra. CVS spectra of acetonitrile display intermolecular coupling of the C≡N mode vibrations, whose cross-correlated out-of-phase signature is evidence for near-perpendicular pair arrangements. Performing a theoretical analysis of the CVS response, an equation for the effective average orientational angle between C≡N groups of adjacent liquid molecules is developed and solved. The effective average orientational angle between adjacent acetonitrile dipoles is ∼102° ± 2°, which is close to a head-to-tail arrangement.

## Graphical abstract

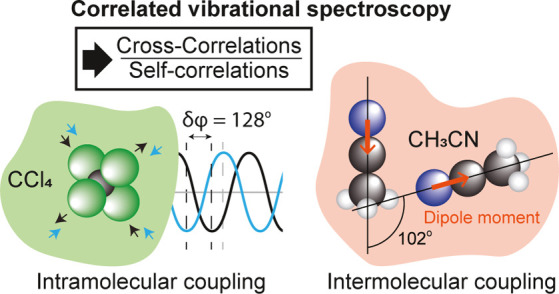

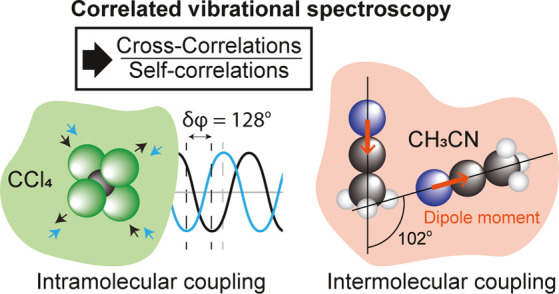

## Introduction

The dynamic molecular structure of a liquid results from a complex balance of interactions that act together with thermal effects. Some of these interactions are highly directional while others are isotropic.^1–5^ Collective dynamic phenomena occur over nanosecond to microsecond time scales and across nanometer to micrometer length scales, resulting in significant heterogeneity.^6–8^ Further complexity arises from quantum mechanical effects, as well as polarization and charge transfer interactions.^9–12^ Understanding the structure and the specific intermolecular interactions of liquids therefore remains challenging.

One of the most successful experimental methods to obtain insight into liquid structure is vibrational spectroscopy,^13^ which enables the direct interrogation of molecular groups via their specific vibrational frequencies. Vibrational spectroscopy includes infrared (IR)/terahertz (THz) and Raman scattering spectroscopy.^14^ Although both these methods measure vibrational modes, one important difference between them is the selection rule; dipole transition moments determine IR/THz transitions, while changes in the polarizability determine Raman modes. Another difference is the method of excitation and the detection configuration. IR/THz spectroscopy rely on direct excitation with IR/THz radiation, which necessitates the use of specific light sources and detectors. For Raman and hyper-Raman scattering (HRaS), excitation and detection are done at visible frequencies, which enables the recording of a broad spectral range. For both methods an intensity spectrum *I*(ω) is measured. *I*(ω) arises from the macroscopic polarization (***P***) in the sample. ***P*** represents the light-matter interaction, and is the sum of the molecular polarizabilities of all molecules (ν, ν′). *I*(ω), ***P,*** and ***p***_ν_ are related by time-correlation functions:

$$I\left(\omega \right)\propto \int_{0}^{\infty }dte^{-i\omega t}\frac{d}{dt}\left\langle P\left(0\right)\cdot P\left(t\right)\right\rangle$$ 1

$$\left\langle P\left(0\right)\cdot P\left(t\right)\right\rangle =\sum_{\nu =1}^{N}\left\langle p_{\nu }\left(0\right)\cdot p_{\nu }\left(t\right)\right\rangle +\sum_{\nu =1}^{N}\left\langle p_{\nu }\left(0\right)\cdot \underset{\nu^{\prime }\neq \nu }{\sum }p_{\nu^{\prime }}\left(t\right)\right\rangle$$ 2

*I*(ω) is composed of self-correlations (SC, first term, eq 2) and cross-correlations (CC, second term, eq 2) of the molecular polarizability ***p*** *_ν_*. The SC term represents the averaged molecular response of single molecules. The CC term represents the molecular response of interacting molecules. Intensity recordings used for THz/IR and Raman spectroscopy follow eq 1 and do not distinguish between information arising from noninteracting single molecules in a structurally averaged environment or from interacting molecules. In order to nonetheless obtain such information, pump–probe techniques,^15–17^ and related methods, such as photon-echo,^18^ 2D-IR,^19^ 2D IR THz,^20^ 2D THz Raman^21^ circumvent some of these aspects.^22–25^ These methods access information about averaged orientational dynamics, or the coupling of certain vibrational modes by exciting at a particular frequency/frequency range and mapping the energy redistribution by recording the changes in the IR or Raman spectrum. Further information about intermolecular interactions is then extracted with the help of MD simulations.

We recently developed a method to separate the spectral contributions of interacting molecules from single-molecule ones using a symmetry based approach. This method, correlated vibrational spectroscopy (CVS),^12^ is a vibrational spectroscopic technique that makes use of the hyper-Raman effect. When an near-infrared or visible laser pulse is focused in a liquid (Figure 1A), second-harmonic and hyper-Raman photons are generated,^26^ via the energy level diagrams in Figure 1B. The second-harmonic (SH) photons emerge from an elastic scattering process, which is the second-order equivalent of linear Rayleigh scattering, while the hyper-Raman photons emerge from an inelastic process whereby the molecule is left in a vibrationally excited state (Stokes HRaS). This is the nonlinear equivalent of Raman scattering, and the selection rule for HRaS is a change in the second-order hyperpolarizability, which happens for all IR-allowed transitions, for most Raman transitions, and additionally for modes that are silent in both IR and Raman spectroscopy.^27^ Recently, interest in HRaS has been renewed, taking advantage of the different selection rules of HRaS to access vibrational modes that are silent in IR and/or Raman spectroscopy.^28,29^ Furthermore, HRaS spectra of various liquids (H_2_O, D_2_O, DMSO, benzene, pyridine, CCl_4_, acetone, acetonitrile, chloroform and others) have been reported.^28,30–36^ HRaS also suffers from the disadvantages of eqs 1 and 2. CVS solves this issue by using the nonlinearity of the light-matter interaction process in combination with the spatial symmetry of the liquid. This is achieved by using a polarimetric decomposition (Figure 1C) derived from nonlinear light scattering symmetry selection rules.^12^ Measuring HRaS spectra at specific scattering angles and in specific polarization combinations allows one to obtain separate spectra for the SC and CC contributions, thereby separately measuring vibrational spectra of noninteracting (single) and interacting (orientationally correlated) molecules. As an example, Figure 1D shows the SC (black) and CC (blue) spectra of neat water. The H-bond stretch mode in the CC spectrum (∼205 cm^–1^), which uniquely arises from interacting water molecules, was used to measure nuclear quantum effects in liquid water, and to determine the amount of charge transfer between hydrated protons and hydroxide ions with the H-bond network.^12^

**Figure 1:**
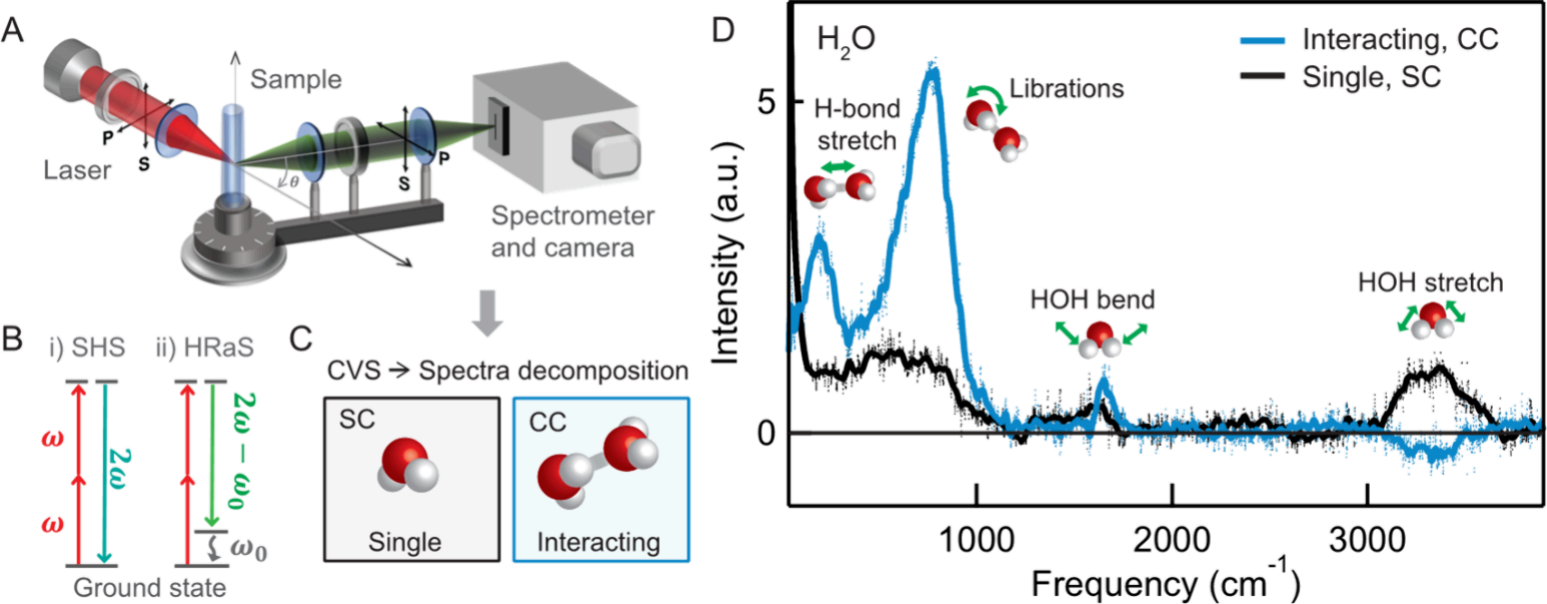
Correlated vibrational spectroscopy (CVS), adapted from ref 12. (A) Experimental setup. S-polarized light is defined as light polarized perpendicular to the scattering plane; P-polarized light is defined as polarized parallel to the scattering plane. In CVS, polarization selection rules are used to decompose the spectra into SC and CC contributions. (B) Energy level diagrams of (i) nonresonant second-harmonic scattering (SHS), which produces a photon at the double frequency, 2ω, and (ii) hyper-Raman scattering (HRaS), where a photon at frequency 2ω – ω_0_ is generated. (C) Illustration of the SC and CC contributions. Spectra are collected and decomposed into *I*_*SC*_ and *I*_*CC*_ contributions using eqs 11a and 11b. (D) CVS spectra of liquid water: vibrational modes involving multiple water molecules appear in the CC spectrum (H-bond stretch, collective pairwise librations), whereas single molecule vibrations are retrieved in the SC response.

Here, we apply CVS to investigate intra- and intermolecular interactions in two liquids – CCl_4_ (carbon tetrachloride) and CH_3_CN (acetonitrile) – as case studies. Analytical calculations reveal that comparing SC and CC vibrational mode intensities provides quantitative insights into the relative phases of vibrational modes and the relative orientations of interacting pairs of molecules. For CCl_4_, CVS spectra display no intermolecular coupling, consistent with its isotropic and short-range intermolecular forces. However, strong intramolecular coupling is observed at the Fermi resonance, and the relative phase between the correlated vibrational modes is determined from the spectra. The CVS spectra of acetonitrile display both intra- and intermolecular coupling via the C≡N mode vibrations. Additionally, the out-of-phase signature of the C≡N mode was used to compute the effective average orientational angle between C≡N groups, which is 102 ^o^ ± 2°. This angle is consistent with ‘head-to-tail’ pair arrangements previously predicted from computations.

## Theoretical Considerations

### Background of Correlated Vibrational Spectroscopy

In a hyper-Raman process (depicted in the energy level diagram of Figure 1B), two photons with the same frequency ω and wavenumber ***k***_1_ interact with the molecules in the medium. The electromagnetic field of the incident laser beam felt by a molecule *v* at position ***r***_υ_ is defined as ***Ẽ*** *_v_* (ω) ≡ ***E*** (ω)*e* ^*i* ***k***_1_ ***· r***_υ_^ + *c*. *c*., and ***E*** (ω) = ***E***_0_ *e* ^−*i*ω*t*^. Second-harmonic scattering (SHS) corresponds to an elastic scattering process, where the fundamental photons are converted into a photon with wavevector ***k***_0_ and a double frequency 2ω, which is scattered at an angle θ with respect to the incoming beam. Conversely, HRaS corresponds to an inelastic process, where the photons are converted into a Stokes hyper-Raman photon with frequency 2ω – ω_0_, whereby ω_0_ is related to the vibrationally excited state (having energy *ℏ*ω_0_, see Figure 1B) in which the molecule is left after emission. In analogy to Raman scattering there is also anti-Stokes hyper-Raman emission.

The incoming field induces a molecular polarizability ***p***_*v*_^(2)^, and the total macroscopic polarizability ( $\underset{v}{\sum }p_{v}^{\left(2\right)}$ ) is responsible for the emission of light having an intensity (*I*):

$$I∼{\left|\sum_{v}p_{v}^{\left(2\right)}\right|}^{2}=\left\{\overset{\underset{v}{\sum }{\left|p_{v}^{\left(2\right)}\right|}^{2}}{\underset{SC\;term}{︸}}+\overset{\underset{{v\neq v}^{\prime }}{\sum }p_{v}^{\left(2\right)}p_{v\prime }^{*\left(2\right)}e^{iq\cdot \left(r_{\upsilon }-{r\prime }_{\upsilon \prime }\right)}}{\underset{CC\;term}{︸}}\right\}=I_{SC}+I_{CC}$$ 3

The first term in eq 3 represents self-correlations of individual molecules, and leads to an incoherent contribution to the total intensity (*I*_*SC*_). This is incoherent light scattering. This type of emission is present for every molecule that has an anisotropic structure. The second term in eq 3, i.e., the double summation over *v* and *v*′, represents cross-correlations between different molecules and leads to a coherent contribution to the total intensity (*I*_*CC*_). The correlation between two different molecules also has a phase factor, *e* ^*i* ***q ·*** (***r***_υ_ ***– r***’_υ’_)^, in which ***q ·*** (***r***_υ_ ***– r***’_υ’_) is the phase difference between the emitted field of the two molecules, and ***q ≡ k***_0_ – 2 ***k***_1_, the scattering wave vector. The second term brings insight into the liquid structure, as it reports on the orientational correlations of the molecules and therefore on the interactions between different molecules. The emission is thus composed of self-correlations (SC term, *I*_*SC*_) and cross-correlations (CC term, *I*_*CC*_) just like in eq 2. These two types of contributions are illustrated in Figures 1C/1D, showing the decomposed CVS spectrum of liquid water.

To explicitly consider the different frequencies we examine the molecular hyperpolarizability, **β**_ν_^(2)^, which determines the molecular polarization together with the incoming fields, via:

$$p_{v}^{\left(2\right)}≡\beta_{v}^{\left(2\right)}:E_{v}E_{v}$$ 4

In hyper-Raman scattering, the polarizability is determined by the static second-order hyperpolarizability tensor (**β**_0, ν_^(2)^) together with an extra derivative term for each vibrational mode *l*. In analogy to Raman and IR spectroscopy, this extra term is the derivative of the hyperpolarizability along the coordinate of the vibrational mode ( $\beta_{v}^{(2)\prime }={\left(\frac{\partial \beta_{\nu }^{\left(2\right)}}{\partial Q_{l}}\right)}_{0}$ ), and the selection rule for a mode to be hyper-Raman active is that ${\left(\frac{\partial \beta_{\nu }^{\left(2\right)}}{\partial Q_{l}}\right)}_{0}\neq 0$ . The total hyperpolarizability for a molecule with *n* vibrational modes is^37,38^

$$\beta_{\nu }^{\left(2\right)}=\beta_{0,\nu }^{\left(2\right)}+\sum_{l=1}^{n}{\left(\frac{\partial \beta_{\nu }^{\left(2\right)}}{\partial Q_{l}}\right)}_{0}Q_{l}=\beta_{0,\nu }^{\left(2\right)}+\sum_{l=1}^{n}\beta_{v}^{(2)\prime }Q_{l}$$ 5

where *Q* *_l_* is the normal mode coordinate of vibration *l*, with resonance frequency ω_0, l_. The ‘0’ subscript for the partial derivative indicates that it is determined at the equilibrium position. The molecular polarization then becomes

$$p_{v}^{\left(2\right)}≡\left(\beta_{0,\nu }^{\left(2\right)}+\sum_{l=1}^{n}\beta_{v}^{(2)\prime }Q_{l}\right):E_{v}E_{v}$$ 6a

$$p_{v}^{\left(2\right)}≡\beta_{0,\nu }^{\left(2\right)}:E_{v}E_{v}+\sum_{l=1}^{n}\beta_{v}^{(2)\prime }Q_{l}:E_{v}E_{v}$$ 6b

Writing this out in the polarization directions *i*, *j*, and *k*, with a normal mode displacement *Q*_*l*_ = *Q*_0,*l*_cos­(ω_0,l_ *t*), and the incoming field as defined above, the full nonlinear source polarization *p*_*v*, *i*_^(2)^ is given by^27,38^

$$p_{\nu ,i}^{\left(2\right)}\left(t\right)=\frac{1}{4}\beta_{\nu ,ijk}^{0}E_{0j}E_{0k}\cos\left(2\omega \right)t+\frac{1}{4}\beta_{\nu ,ijk}^{0}E_{0j}E_{0k}+\frac{1}{8}\sum_{l=1}^{n}\left(2{\mathrm{\beta ̇}}_{\nu ,ijk}^{\left(2\right)}Q_{0,l}E_{0j}E_{0k}\cos\left(\omega_{0,l}\right)t+{\mathrm{\beta ̇}}_{\nu ,ijk}^{\left(2\right)}Q_{0,l}E_{0j}E_{0k}\cos\left(2\omega -\omega_{0,l}\right)t+{\mathrm{\beta ̇}}_{\nu ,ijk}^{\left(2\right)}Q_{0}E_{0j}E_{0k}\cos\left(2\omega +\omega_{0,l}\right)t\right)$$ 7

This expression shows that the molecular polarization oscillates at several frequencies: a second harmonic frequency (1st term), a DC static field from optical rectification (2nd term), an IR frequency (3rd term), and Stokes/anti-Stokes hyper-Raman frequencies (4th and 5th terms, respectively). The Stokes HRaS term, which peaks at 2ω – ω_0, *l*_, is the one relevant for CVS. The modes that are HRaS allowed for H_2_O, CCl_4_ and CH_3_CN are as following: For H_2_O with C_2v_ symmetry, all modes are allowed. For CCl_4_, with *T*_d_ symmetry, A_1,_ F_1,_ and F_2_ modes are allowed.^39,40^ For CH_3_CN, with C_3v_ symmetry, all *a* and *e* vibrational modes are allowed.^34,41^ The intensity (eq 3) at ω' can be found using the solution for the molecular polarization (eq 6a or 7), and it becomes

$$I\left(\omega^{\prime }\right)\propto \sum_{l=1}^{n}\left\langle {\left|u\cdot p_{\nu }^{\left(2\right)}\left(\omega^{\prime }\right)\right|}^{2}\right\rangle$$ 8

where ***u*** is the polarization state of the outgoing beam and ω' = 2ω – ω_0_. The brackets ⟨⟩ represent an ensemble orientational average over all involved molecules and over the time duration of the laser pulse.

In order to find an analytical solution, we assume that the liquid is isotropic.^37^ As a consequence, all the cross-correlation terms vanish. Since these terms correspond to interacting molecules, they are not present in the analytical solution. Equation 8 can be further developed by inserting eq 7, and taking only the hyper-Raman frequencies into account, this becomes

$$I\left(\omega^{\prime }\right)\propto \left\langle E_{0}^{4}\overset{\underset{ijk}{\sum }\underset{{ij\prime k\prime }^{\prime }}{\sum }u_{i}v_{j}v_{k}u_{i\prime }v_{j\prime }v_{k\prime }\beta_{v,ijk}^{\left(2\right)\prime }\beta_{v,i\prime j\prime k\prime }^{\left(2\right)\prime }}{\underset{\mathrm{average}\;\mathrm{over}\;\mathrm{lab}\;\mathrm{frame}\;\mathrm{rotations}}{︸}}\overset{{\left|\overset{\sim }{Q}\left(\omega^{\prime }\right)\right|}^{2}}{\underset{\mathrm{average}\;\mathrm{over}\;\mathrm{normal}\;\mathrm{mode}\;\mathrm{displacements}}{︸}}\right\rangle$$ 9

where *Q̃*(ω′) is defined as the Fourier transform of *Q*(*t*), as *Q̃*(ω′) = ∫ *Q*(*t*)*e* ^−*i*ω′*t*^d*t*, the unit vector ***v*** represents the polarization direction of the incoming electric field, and the unit vector ***u*** represents the polarization direction of the outcoming electric field. Equation 9 can be separated into a product of two averages: The average over all possible orientations of the molecules, which affects only the hyperpolarizability term, and the average over all possible initial displacements (*Q*_*l*_(0)), which affects only the normal-mode term.

The second term becomes a Lorentzian, which is insensitive to the spatial arrangement of molecules in the liquid. The first term in the product of eq 9, can be computed using coordinate transformations and averaging them. Flór et al. showed that for an isotropic liquid there are symmetry relations between intensities recorded in the SSS, PPP, SPP, and PSS (out, in, in) polarization combinations (with S (P) being perpendicular (parallel) to the horizontal plane of scattering, see Figure 1).^12^ These intensity relations are the same as those previously derived for SHS:^42^

$$I_{SPP}=I_{PSS}$$ 10a

$$I_{PPP}-\cos^{2}\left(\theta \right)I_{SSS}-\sin^{2}\left(\theta \right)I_{SPP}=0$$ 10b

In addition, for a near-isotropic arrangements, cross-correlations are detected only when the polarization direction of the emitted light (***u***) has a parallel component with the scattering wavevector (***q***).^12,43^ This means that the SSS and SPP combinations are entirely insensitive to cross-correlations, and only probe self-correlations. Conversely, PPP is sensitive to cross-correlations. When cross-correlations are present in the sample, eq 10b provides a means to measure them. Cross- (*I*_*CC*_) and self-correlated (*I*_*SC*_) intensities can be separately measured noting that

$$I_{SC}=I_{SSS}+I_{SPP}$$ 11a

$$I_{CC}\left(\theta \right)=I_{PPP}-\cos^{2}(\theta )I_{SSS}-\sin^{2}(\theta )I_{SPP}$$ 11b

### CVS Compared to Other Methods

Figure 1D illustrates the application of CVS to liquid water. The black *I*_*SC*_ spectrum is found by measuring HRaS spectra in the SSS and SPP polarization combinations. The blue *I*_*CC*_ spectrum is created by measuring PPP, SSS, and SPP spectra at a scattering angle that is most sensitive to picking up cross-correlations (here, chosen to be θ = 15°), and applying eq 11b. Collective vibrational modes involving multiple water molecules – such as the H-bond stretching mode at ∼ 205 cm^–1^ and the collective librational modes between 400–1000 cm^–1^ – are present in the CC spectrum but mostly absent from the SC spectrum. In contrast, localized intramolecular vibrations, such as the O–H stretching modes, exhibit only weak CC contributions and are primarily found in the SC spectrum. Notably, the case of water reveals a distinction between the different spectroscopic techniques: Raman spectra closely resemble the SC spectrum, while IR spectra show a stronger similarity with the CC spectrum.^12^ This observation indicates that Raman spectroscopy predominantly probes local vibrations through incoherent emission, whereas IR absorption spectroscopy is more sensitive to collective effects via fluctuations in the dipole moment.^44^ Conversely, hyper-Raman scattering – the process underlying CVS – is sensitive to both local and collective vibrational effects, as reflected in its SC and CC contributions. Moreover, hyper-Raman scattering offers a broader set of selection rules compared to IR and Raman spectroscopy alone. While IR absorption requires a change in dipole moment along the vibrational coordinates and Raman scattering requires a change in polarizability, hyper-Raman scattering depends on a change in the second-order hyperpolarizability. This criterion will always reflect changes in the dipole moment and often also in the polarizability, meaning that, in general, IR- and Raman-active modes also appear in the hyper-Raman spectrum.^45^ Thus, CVS combines the advantages of both IR and Raman techniques while also uniquely enabling the separation of single (SC) from interacting (CC) contributions.

Next, we further develop the application of CVS, namely the quantification of intramolecular Fermi resonances and the use of the cross-correlated spectrum to determine the relative orientational angle between a pair of molecules. The full theoretical analysis is provided in the Appendix. In the Results and Discussion section we will use this analysis to understand a nonpolar liquid, CCl_4_ (intramolecular coupling) and a polar one, acetonitrile (intermolecular coupling).

## Methods

### Chemicals

Carbon tetrachloride (CCl_4_, 99.9%, Sigma-Aldrich) and acetonitrile (CH_3_CN ≥ 99.9%, Sigma-Aldrich) were used as received without further purification.

### CVS

Hyper-Raman scattering (HRaS) spectra were recorded using a Yb:KGW laser (Pharos-SP system) at a 200-kHz repetition rate combined with a 1032 nm bandpass filter (15–401, Edmund Optics). The resulting pulse duration was centered at 1033 nm, had a 4 ps FWHM time duration and a 25 cm^–1^ spectral FWHM. The polarization of the input pulses was controlled by a Glan-Taylor polarizer (GT10-B, Thorlabs) in combination with a zero-order half-wave plate (WPH05M-1030). The filtered (FEL0750, Thorlabs) input pulses had a pulse energy of 0.42 μJ (incident laser power P = 85 mW). The beam was focused into a cylindrical glass sample cell (inner diameter 4.2 mm) with a beam waist diameter of ∼ 80 μm. HRaS spectra were collected at a scattering angle of θ = 15°. To do so, an achromatic doublet (f = 5 cm, AC127–050-A-ML, Thorlabs), oriented at 15° from the fundamental beam in the scattering plane, was used. A 12 mm-diameter iris was employed to limit the acceptance angle to 7°. The output light was polarization-analyzed by combining of a zero-order half-wave plate (WPH05M-514, Thorlabs) and a Glan-Taylor polarizer (GT10-A, Thorlabs). The latter was used to rotate the selected polarization parallel to the entrance slit of the subsequent spectrometer, to ensure that the polarization dependence of the spectrometer does not impact the measurements. The analyzed light was focused onto the entrance slit of a spectrometer (Princeton Instruments Acton SP2300) using a plano-convex lens (f = 5 cm, LA1255-A, Thorlabs), and spectrally dispersed with a 1200 g/mm blazed grating (500 nm) onto an intensified charge coupled device (iCCD) camera (PiMax 4, Princeton Instruments). The spectrometer and camera were calibrated using the Teledyne IntelliCal system. The camera was positioned using the spectral lines of an argon wavelength calibration lamp. The integration time for each spectrum is 50 s, with an average being collected over up to 15 exposures. All the measurements were performed in a temperature- and humidity- controlled room (T = 297 K; relative humidity, 26.0%). The experimental setup is sketched in Figure 1B. SC and CC spectra were obtained by recording spectra with multiple polarization combinations and applying eqs 11a and 11b.

## Results and Discussions

### CVS Spectra of CCl_4_

#### Intramolecular Coupling

Nonpolar liquids are composed of molecules with a zero average permanent dipole moment. Interactions between nonpolar molecules are mainly driven by weak, short-ranged dispersive forces, which act primarily on the first solvation shell.^2^ Nonpolar molecules are often represented using a Lennard-Jones potential, with negligible intermolecular correlations (referred to as a noninteracting liquid in the following). Room temperature CCl_4_ is an example of such a liquid, with a tetrahedral geometry belonging to the *T*_d_ point group. IR, Raman, and hyper-Raman spectra of CCl_4_ have been previously published in refs. 33,39,40,45–47 For a noninteracting liquid, all stretching, bending and scissoring modes are uncorrelated and are thus expected to be part of the SC spectrum. Figure 2A shows the measured CVS spectra, with the SC and CC spectrum in black and blue, respectively. The center frequency of the SHS peak at 515 nm (19417 cm^–1^) is taken as the 0 cm^–1^ shift. The SC–CVS spectrum is shown in Figure 2A, and shows the CCl_4_ scissoring mode (δ_scis_ (ν_2,_ E), 222 cm^–1^), the bending mode (δ_bend_ (ν_4_, F_2_), 314 cm^–1^), and the symmetric C–Cl stretch mode (ν_s_ (ν_1,_ A_1_), 454 cm^–1^). Note that the ν notations here refer to vibrational modes and should not be confused with the notation ν for molecules. The CCl_4_ CC spectrum contains none of the above vibrational modes, as expected, highlighting that CCl_4_ is an isotropic liquid with no orientation cross-correlations, due to short-ranged isotropic intermolecular bonding. Interestingly, above 700 cm^–1^, a vibrational mode appears at ∼ 767 cm^–1^, that is around 7 times more intense than the other modes, and contributes not only to the SC spectrum but also to the CC spectrum. This mode has been measured before in nanosecond HRaS^33,40,45^ and was suggested to be due to either an isolated single molecule mode,^40^ to polaritons (that is, couplings between the electromagnetic field and a wave of excited dipoles),^45^ or to long-range dipole–dipole coupling over distances >200 nm.^33^

**Figure 2:**
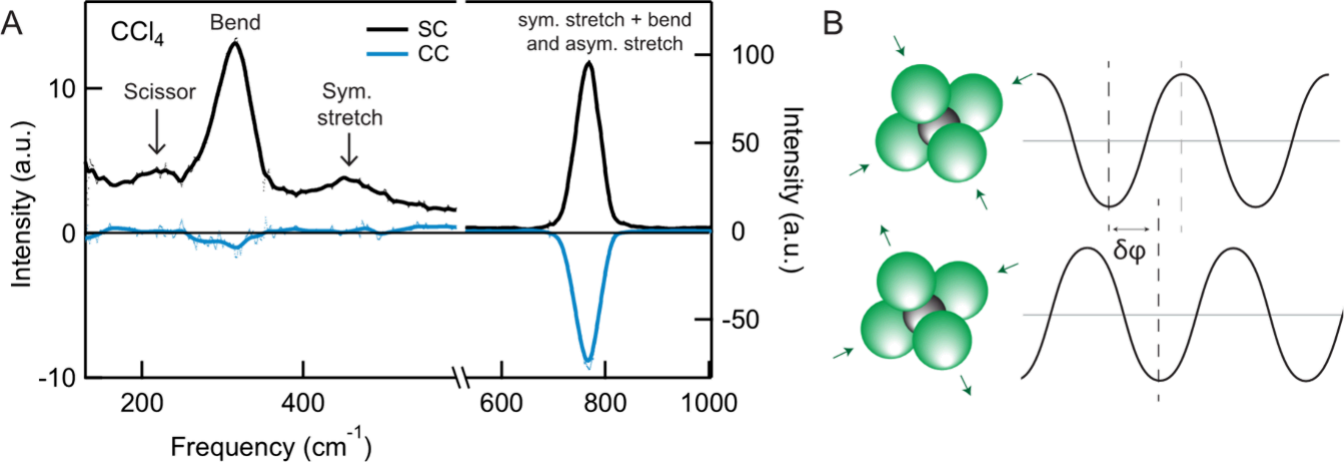
CVS of liquid CCl_4_: intramolecular coupling. (A) CVS SC (black) and CC (blue) spectra of CCl_4_. The scissoring mode (ν_2_), the bending mode (ν_4_) and the symmetric C–Cl stretch mode (ν_1_) do not contribute to the CC spectrum, confirming that the liquid is isotropic with noninteracting molecules. The peak at ∼767 cm^–1^ is an exception, showing an unusually high intensity and a strong negative CC contribution. It arises from intramolecular coupling, by means of a Fermi resonance between the ν_1_ + ν_4_ combination with the asymmetric C–Cl stretch vibration (ν_3_). (B) Illustrations of these coupled modes, and their phase difference as determined from the CC/SC intensity ratio.

Inspecting the spectral mode at ∼ 767 cm^–1^ in more detail, it is actually composed of two modes, that are both IR and Raman active and have overlapping frequencies: the combination band of a symmetric C–Cl stretch mode with the C–Cl bending mode (ν_1_ + ν_4_) at 762 cm^–1^, and the asymmetric C–Cl stretch mode (ν_3_) at 786 cm^–1^ (IR), and 791 cm^–1^ (Raman).^46,47^ As these vibrations have the same symmetry, the ν_3_ mode is in Fermi resonance with the ν_1_ + ν_4_ combination band, and thus there is a very strong correlation between these two vibrational modes. Note that the peaks are convoluted here, with broader ps-pulses in Figure 2A, but appear as separate and well-defined in ns experiments.^48^ These two distinct vibrational modes oscillate on the same molecule and are thus coupled. This also means there is a fixed but unknown phase difference between them.^13^ It is this coupling that manifests itself as the strong peak in the SC/CC spectrum: Two different vibrational modes on the same molecule should lead to an extra coupling term to eq 3. Equation 3 contains the CC term, which refers to orientational cross-correlations of a specific vibrational mode *l* on molecule ν, with the same *l* mode on another molecule ν′. In the case of a fortuitous overlap of two different vibrational modes *l* and *l*′ on the same molecule ν with the same frequency, an additional intramolecular correlation appears. Explicitly including such vibrational mode coupling in eq 3, we obtain

$$I∼\left\{\underset{\mathrm{SC}\;\mathrm{term}}{\underset{︸}{\underset{v,l}{\sum }|p_{v}^{\left(2\right)}\left(2\omega -\omega_{0,l}\right){|}^{2}}}+\underset{\mathrm{inter}\mathrm{molecular}\;\mathrm{coupling}\;\left(\mathrm{CC}\right)}{\underset{︸}{\underset{v\neq v\prime }{\sum }p_{v,l}^{\left(2\right)}\left(2\omega -\omega_{0,l}\right)p_{v^{\prime },l}^{*\left(2\right)}\left(2\omega -\omega_{0,l}\right)e^{iq\cdot \left(r_{\upsilon }-r_{\upsilon^{\prime }}^{\prime }\right)}}}+\underset{\mathrm{intra}\mathrm{molecular}\;\mathrm{coupling}}{\underset{︸}{\underset{l\neq l\prime }{\sum }p_{v,l}^{\left(2\right)}\left(2\omega -\omega_{0,l}\right)p_{v,l^{\prime }}^{*\left(2\right)}\left(2\omega -\omega_{0,l^{\prime }}\right)e^{i\varphi }}}\right\}$$ 12

whereby ω_0, *l*_ ∼ ω_0, *l*′_ or ω_0, *l*_ = ω_0, *l*′_. The phase φ is the phase difference between the two modes, and if we assume that the spectral line shape is the same for both modes, the phase φ can be retrieved by comparing the relative intensities of the CC and SC spectra. To this end, the single-molecule response is considered in the molecular frame, with two emission sources which are the resonant vibrational modes. The two modes thus emit in the same frequency region, and we can write the sum of self- and cross-correlations as $I_{SC}+I_{CC}=I_{v_{1}+v_{4}}+I_{v_{3}}+2\sqrt{I_{v_{1}+v_{4}}I_{v_{3}}}\cos\;\varphi$ where the first term *I*_ν_1_+ν_4__ + *I*_ν_3__ represents the SC contribution and $2\sqrt{I_{v_{1}+v_{4}}I_{v_{3}}}\cos\;\varphi$ the CC contribution, *I*_ν_i__ representing the respective intensities. The SC peak is fitted with two Lorentzian functions to retrieve *I*_ν_1_+ν_4__ and *I*_ν_3__. Subsequently, these values are used to fit the measured CC response with $2\sqrt{I_{v_{1}+v_{4}}I_{v_{3}}}\cos\;\varphi$ , to retrieve the phase φ. This yields a value of φ = 128 ± 3°. In other words, the Fermi-resonant vibrational modes are anticorrelated with a phase difference of 128 ± 3°, as sketched in Figure 2C. CVS thus represents a unique approach to analyze intramolecular vibrational coupling.

### CVS Spectra of Acetonitrile

#### Intermolecular Coupling

Next, we investigate a case of intermolecular coupling in liquid acetonitrile (CH_3_CN, Figure 3A), which has a strong C≡N dipole moment of 3.92 D,^49^ and is predicted by computations to have a strong pairwise intermolecular coupling.^50–52^ The behavior of acetonitrile in the liquid phase has been extensively studied.^49,51–61^ Acetonitrile displays intramolecular coupling, which is due to vibrational energy levels of acetonitrile exhibiting significant anharmonicity.^41^ The structure of liquid acetonitrile is primarily governed by strong dipole–dipole interactions between the C≡N groups.^55,61^ Pure dipole–dipole coupling might be thought to give rise to an antiparallel arrangement of molecules.^59,60^ However, recent studies^50,62^ in which neutron scattering and X-ray diffraction measurements are interpreted using MD simulations suggest that the structure is more complex but still predominantly determined by pairwise interactions that give rise to head-to-tail complexation.

**Figure 3:**
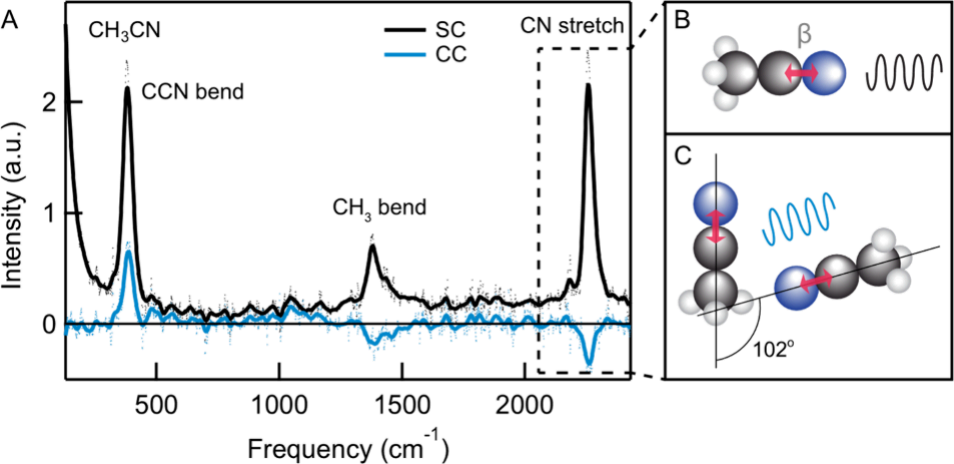
CVS spectra analysis in acetonitrile. (A) CVS SC (black) and CC (blue) spectra of liquid acetonitrile. Among the vibrational modes identified in the spectrum, the C≡N stretch mode is used to estimate the intermolecular angle. (B) Sketch of the C≡N stretch vibrational mode together with the direction in which the second-order hyperpolarizability changes most. (C) Sketch of a pair of acetonitrile molecules whose dipole moments are under an angle of 102°. This angle influences the phase of the peak in the CC spectrum, which is negative.

Figure 3A shows the CVS spectra of liquid acetonitrile, with the SC and CC spectrum in black and blue, respectively. The SC spectrum contains the following prominent vibrational modes: the CCN bending mode at ∼ 378 cm^–1^, the sym. and asym. CH_3_ bending modes between ∼ 1378–1430 cm^–1^ and the C ≡ N stretch mode at ∼ 2255 cm^–1^. These peaks have been observed before in HRaS experiments recorded at a 90° scattering angle and with S-polarized detection.^34,41^ These spectra agree with the SC spectrum in Figure 3A. The CC spectrum (blue) contains the CCN bending mode (383 cm^–1^, positive), the CH_3_ bending mode (1378–1430 cm^–1^, negative) and the C≡N stretch mode (∼2259 cm^–1^, negative). The negative CC response for the CH_3_ bending mode feature is associated with the presence of a previously observed Fermi resonance between the fundamental ν_3_ mode (CH_3_ bending mode) and the combination band ν_7_+ν_8_ (CH_3_ rocking and torsion).^53,55^ This is similar to the intramolecular coupling observed in CCl_4_. The CCN bending mode and the C≡N stretch mode represent dipole moment changes that do not have intramolecular coupling. Their cross-correlation responses therefore represent intermolecular couplings between the different vibrational modes.

For the C≡N stretch mode, a strong negative CC intensity is observed (Figure 3A, highlighted by the dashed box). We investigate this in more detail by performing a theoretical analysis to extract the relative angle between molecules. If there is an intermolecular coupling in liquid acetonitrile, the vibrational modes of the groups that participate in this coupling should be visible in the CC spectrum, while the type of molecular arrangement should impact the *I*_*CC*_/*I*_*SC*_ ratio. From eqs 11a and 11b it is possible to obtain information about the averaged relative molecular orientation between groups, based on the values of the hyperpolarizability derivatives of the vibrational mode of interest, together with the distance between the groups, the number of interacting groups and the type of interaction between them. The full analysis is given in the Appendix, and shows that (1) the *I*_*SC*_ spectrum is determined by the derivative values of the second-order hyperpolarizability that can be computed numerically; (2) The *I*_*CC*_ spectrum is determined by the same derivative values, but these are coupled to the relative molecular orientation, whose average depends on the interaction free energy. (3) For a dipole-type interaction the *I*_*CC*_/*I*_*SC*_ ratio for the C≡N stretch mode can be expressed in terms of the average tilt angle (θ) between the dipole moments of a pair of acetonitrile molecules (sketched in Figure 3C). Equation [Disp-formula eqA15] shows that the $\frac{I_{CC}}{I_{SC}}$ ratio can be written as

$$\frac{I_{CC}}{I_{SC}}=0.458\left(\left\langle \cos\;\theta \right\rangle +0.8\left\langle \cos^{3}\;\theta \right\rangle \right)$$ 13

whose measured value is −0.16 (Figure 3A). Solving this numerically, we obtain ⟨cos θ⟩ = – 0.22, and determine the effective average orientational angle, corresponding to an effective angle of 102° ± 2°, where error bars are given based on the experimental noise. Since the CCN bending mode is related in orientation to the C≡N stretch mode (with a 90° rotation), the average angle should be related, which on average becomes ∼ 75°. This should give rise to a positive contribution to the CC spectrum and this is indeed observed in Figure 3A.

Thus, the CVS spectra of liquid acetonitrile provide direct evidence of intra- and intermolecular coupling, and the averaged orientational angle between pairs of acetonitrile molecules agrees with predictions from MD simulations that show substantial populations of ‘head-to-tail’ dimers.^50^ This type of detailed information about interactions/orientational distributions was previously not accessible experimentally, as other spectroscopies cannot distinguish between single molecules and interacting molecules. Molecular orientation is accessible on interfaces by means of vibrational sum frequency generation, to which the current analysis shares similarities.

## Conclusions

In summary, we have demonstrated the potential of correlated vibrational spectroscopy (CVS) to investigate intra- and intermolecular interactions in liquids, using carbon tetrachloride (CCl_4_) and acetonitrile (CH_3_CN) as case studies. By separately analyzing self-correlated (SC) and cross-correlated (CC) vibrational contributions, CVS provides direct access to molecular coupling effects that are challenging to extract using conventional IR and Raman spectroscopies.

For CCl_4_ our results confirm the absence of significant intermolecular interactions, consistent with the isotropic and short-range nature of forces in this nonpolar solvent. However, strong intramolecular coupling is observed in the Fermi resonance, and the relative phase of participating vibrational modes is determined from the CVS spectra. In contrast, for CH_3_CN, intermolecular coupling is detected through the C≡N stretching vibrations, revealing out-of-phase signatures indicative of a near-perpendicular head-to-tail molecular arrangement. Through theoretical analysis, the orientational angle between C≡N groups was quantified, providing molecular-level insight into the liquid structure. The computational analysis further highlights how to describe the cross-correlations in the solution, whereby two main parameters come into play, namely the values of the derivative of the second-order hyperpolarizability tensor of the molecular group of interest, as well as the interaction free energy between them. Here, the interaction free energy was known. However, one might envision a scenario in which CVS can be used to also unravel the type of interactions that lead to a particular outcome, for example when measuring a certain chemical conversion process.

This study further highlights the unique capabilities of CVS in resolving vibrational coupling effects and molecular orientations in pure liquids without requiring complex spectral decompositions or external perturbations. As a model-free approach based on symmetry selection rules, CVS has a great potential. It provides detailed molecular-level information about liquids that existing spectroscopic techniques cannot access.

## Data Availability

The data that support the findings of this study are available from the corresponding author upon reasonable request.

## Appendix

### A.1 Determining Pairwise Intermolecular Coupling/orientational Angles with CVS

Consider a pair of molecules having a particular orientational correlation that gives rise to a relative angle γ, induced by an effective free energy *F*(γ), we can then compute expressions for the CC and SC responses. To do so, we take a pair of acetonitrile molecules, and compute the CVS responses based on the HRaS emission of the C≡N stretching mode (∼2255 cm^–1^). We assume that the positions of the two molecules’ centres of mass are fixed in space, with an unknown relative orientation. There are two molecular frames, one, (*x*, *y*, *z*), for molecule 1 and one for molecule 2, (*x* ′, *y* ′, *z* ′). The relative orientation is described using Euler angles, θ, ϕ, χ, which describe the rotation from one axis system to another,

$$\left(\begin{array}{c} x^{\prime }\\ y^{\prime }\\ z^{\prime } \end{array}\right)=\Phi \left(\begin{array}{c} x\\ y\\ z \end{array}\right)$$
A1

where,

$$\Phi =\left(\begin{array}{ccc} \cos\;\phi \cos\;\gamma \cos\;\chi -\sin\;\phi \sin\;\chi & \sin\;\phi \cos\;\gamma \cos\;\chi +\cos\;\phi \sin\;\chi & -\sin\;\gamma \cos\;\chi \\ -\cos\;\phi \cos\;\gamma \sin\;\chi -\sin\;\phi \cos\;\chi & -\sin\;\phi \cos\;\gamma \sin\;\chi +\cos\;\phi \cos\;\chi & \sin\;\gamma \sin\;\chi \\ \cos\;\phi \sin\;\gamma & \sin\;\phi \sin\;\gamma & \cos\;\gamma \end{array}\right)$$

The angle γ is the angle between the symmetry axes (z and z’) of both molecules and describes the angle between both C≡N dipoles. Further, we assume that the C≡N stretching mode, which represents a displacement along z/z’, is excited from its ground state |0⟩ to its first excited state |1⟩, which is reasonable for a high-frequency vibration, noted *l*. The normal mode coordinate describing this vibration is called *Q*_*l*_. The hyper-Raman spectrum at frequency ω is,

$$I\left(\omega \right)\propto \sum_{v=1}^{2}\sum_{v^{\prime }=1}^{2}\frac{\partial \beta_{UVV}^{\left(2\right)}\left(\upsilon \right)}{\partial Q_{l}}\frac{\partial \beta_{UVV}^{\left(2\right)}\left(\upsilon^{\prime }\right)}{\partial Q_{l}}{\left|\left\langle 1\left|{\mathrm{Q̂}}_{l}\right|0\right\rangle \right|}^{2}e^{iq\cdot r_{vv\prime }}$$
A2

where the sums are taken over both molecules and $\frac{\partial \beta_{UVV}^{\left(2\right)}\left(\upsilon \right)}{\partial Q_{l}}$ is the derivative with respect to the normal-mode coordinate of the second-order hyperpolarizability of the *v* ^*th*^ molecule, where *U* is the polarization direction of the outgoing light and V that of the incoming light. For notational convenience we write $\beta {’}_{UVV}\left(v\right)=\frac{\partial \beta_{UVV}^{\left(2\right)}\left(v\right)}{\partial Q_{l}}$ , where the ’ indicates differentiation with respect to the normal coordinate of interest. This derivative can be computed numerically using finite differences. Working in the reference frame as defined above, this is

$$\beta {’}_{ijk}\left(v\right)=\frac{\beta_{ijk}\left(+\delta Q_{l}\right)-\beta_{ijk}\left(-\delta Q_{l}\right)}{2\delta Q_{l}}$$
A3

with β_*ijk*_(*Q_l_*) the value of the hyperpolarizability element as a function of the normal mode coordinate *Q*_*l*_. With δ*Q*_*l*_ = 0.02 *Å* and computing the β tensors at the CCSD level with the 6-311+G* basis set we obtained the values (in a.u./*Å*) given in Table 1.

**Table 1:** Computed Second-Order Hyperpolarizability Tensor Derivatives for Each Tensor Element of the C≡N Stretch Mode of Acetonitrile, in Atomic Units (a.u.)/Å^a^

β′ element	value	β′ element	value	β′ element	value
xxx	0	yxx	5.0517	zxx	–1.9604
xxy	5.0517	yxy	0	zxy	0
xxz	–1.9604	yxz	0	zxz	0
xyx	5.0517	yyx	0	zyx	0
xyy	0	yyy	–5.0517	zyy	–1.9604
xyz	0	yyz	–1.9604	zyz	0
xzx	–1.9604	yzx	0	zzx	0
xzy	0	yzy	–1.9604	zzy	0
xzz	0	yzz	0	zzz	152.7908

a The symmetry axis is the *z*-axis.

#### Self-Correlation and Cross-Correlation spectra

The matrix element |⟨1|*Q̂*_*l*_|0⟩| in eq [Disp-formula eqA2] is common to all terms, so it can be subsumed into the constant of proportionality. The spectral intensity can be separated into two terms, a self-correlation term *I*_*SC*_ and a cross- correlation term *I*_*CC*_,

$$I_{SC}=\sum_{v}\beta {’}_{UVV}{\left(v\right)}^{2}$$
A4a

$$I_{CC}=\sum_{v\neq v^{\prime }}\beta {’}_{UVV}\left(v\right)\beta {’}_{UVV}\left(v^{\prime }\right)e^{iq\cdot r_{vv\prime }}$$
A4b

The self-correlation intensity is given by the sum of two intensities with different polarization combinations: the SSS intensity, in which the incoming and outgoing polarizations are parallel to each other, and the SPP intensity, in which the outgoing polarization is orthogonal to the incoming polarization:

$$I_{SC}=\sum_{v}\beta {’}_{YYY}{\left(v\right)}^{2}+\sum_{v}\beta {’}_{YXX}{\left(v\right)}^{2}$$
A5

The hyperpolarizability derivatives in the lab frame can be written in terms of those in the molecular frame; e.g., $\beta {’}_{YYY}=\underset{abc}{\sum }\beta {’}_{ijk}Y_{a}Y_{b}Y_{c}$ where *Y*_*a*_ is the projection of the *a* ^*th*^ unit vector in the molecular frame on the *Y* axis in the laboratory frame. *I*_*SC*_ becomes

$$I_{SC}=\underset{v}{\sum }\underset{abcdef}{\sum }\beta {’}_{abc}\beta {’}_{def}\left(Y_{a}Y_{b}Y_{c}Y_{d}Y_{e}Y_{f}+Y_{a}X_{b}X_{c}Y_{d}X_{e}X_{f}\right)$$
A6

which needs to be orientationally averaged if we consider many pairs of molecules in the liquid. The way we choose to interpret this is that each molecule has its orientation fixed in space and the experimental apparatus is rotated, which results in

$$I_{SC}=2\sum_{abcdef}\beta {’}_{abc}\beta {’}_{def}\left(\left\langle Y_{a}Y_{b}Y_{c}Y_{d}Y_{e}Y_{f}+Y_{a}X_{b}X_{c}Y_{d}X_{e}X_{f}\right\rangle \right)$$
A7

Using the values in Table 1, a numerical evaluation yields

$$I_{SC}=7847.62a.u./Å$$
A8

The cross-correlation intensity of two molecules is

$$I_{CC}=2\beta {’}_{UVV}\left(1\right)\beta {’}_{UVV}\left(2\right)\cos\left(q\cdot r_{12}\right)$$
A9

If the molecules are neighbors and separated by a small distance cos­(***q·r***_12_) → 1, and

$$I_{CC}=2\beta {’}_{UVV}\left(1\right)\beta {’}_{UVV}\left(2\right)$$
A10

From here, the relative orientation of the two molecules needs to be considered as well. Doing so, the cross-correlation intensity is,

$$I_{CC}=2\underset{abc}{\sum }\underset{def}{\sum }\beta_{\mathrm{abc}}^{\prime }\left(1\right)\beta_{\mathrm{def}}^{\prime }\left(2\right)Y_{a}\left(1\right)Y_{b}\left(1\right)Y_{c}\left(1\right)Y_{d}\left(2\right)Y_{e}\left(2\right)Y_{f}\left(2\right)=2\underset{abcdefghi}{\sum }\beta_{\mathrm{abc}}^{\prime }\beta_{\mathrm{def}}^{\prime }Y_{a}Y_{b}Y_{c}Y_{g}Y_{h}Y_{i}\Phi_{dg}\Phi_{eh}\Phi_{fi}$$
A11

Writing the sum in this way ensures that all of the *Y*_*a*_ factors involve the projection of an axis vector on the first molecule with the laboratory frame, so that all these six factors can be averaged over. The Φ_αγ_ factors account for the transformation between the two reference frames. Assuming that the only correlations between the two molecules are between their dipole moments, we can average over ϕ and χ, and retain the Euler angle γ:

$$I_{CC}=2\underset{abcdefghi}{\sum }\beta_{\mathrm{abc}}^{\prime }\beta_{\mathrm{def}}^{\prime }\left\langle Y_{a}Y_{b}Y_{c}Y_{g}Y_{h}Y_{i}\right\rangle \frac{1}{4\pi^{2}}\int_{0}^{2\pi }\int_{0}^{2\pi }\Phi_{dg}\Phi_{eh}\Phi_{fi}d\phi d\chi$$
A12

This sum contains many terms, most of which are zero. This is due to a combination of the ⟨*Y*_a_ *Y*_b_ *Y*_c_ *Y*_g_ *Y*_h_ *Y*_i_⟩ terms vanishing due to symmetry or because the integral over Euler angles vanishes, while many derivatives are also 0 (Table 1). Evaluating the integrals in eq [Disp-formula eqA12] and inserting the hyperpolarizability derivatives from Table 1 gives,

$$I_{CC}=\left(3592.54\;a\text{.}u\text{.}/Å\right)\cos\;\gamma +\left(2877.35\;a\text{.}u\text{.}/Å\right)\cos^{3}\;\gamma$$
A13

which is the CC response of a pair of molecules whose dipoles have a relative orientation angle γ. Dividing eq [Disp-formula eqA13] by eq [Disp-formula eqA8] gives an expression for the intensity ratio,

$$\frac{I_{CC}}{I_{SC}}=0.458\cos\gamma +0.367\cos^{3}\;\gamma =0.458\left(\cos\gamma +0.8\cos^{3}\;\gamma \right)$$
A14

This is the intensity ratio when the angle between the dipole moments of the two molecules is γ. In reality, this angle will have some probability distribution. Note that in acetonitrile, this distribution is expected to be particularly narrow based on molecular dynamics simulations.^50,62^ The distribution is largely dominated by the effect of neighboring molecules, expected at a distance of ∼ 3.5 Å and an angle of ∼ 180°. The average intensity ratio is

$$\frac{I_{CC}}{I_{SC}}=0.458\left(\left\langle \cos\;\gamma \right\rangle +0.8\left\langle \cos^{3}\;\gamma \right\rangle \right)$$
A15

Which can be solved under the condition that the type of interaction between the molecules is known. With the interaction free energy between the dipoles being *F*(γ), the averages become:

$$\left\langle \cos\left(\gamma \right)\right\rangle =\frac{\int_{0}^{\pi }\cos\;\gamma e^{-\beta F\left(\gamma \right)}\sin\;\gamma d\gamma }{\int_{0}^{\pi }e^{-\beta F\left(\gamma \right)}\sin\;\gamma d\gamma },\;\mathrm{and}\;\left\langle \cos^{3}\;\gamma \right\rangle =\frac{\int_{0}^{\pi }\cos^{3}\;\gamma e^{-\beta F\left(\gamma \right)}\sin\;\gamma d\gamma }{\int_{0}^{\pi }e^{-\beta F\left(\gamma \right)}\sin\;\gamma d\gamma }$$
A16

with β=1kT . Assuming the potential of mean force for the relative orientation of the two molecules is similar to the potential energy of dipole ordering, we have β*F*(γ) = α cos γ. In that case:

$$\left\langle \cos\;\gamma \right\rangle =L_{1}\left(\alpha \right)≡\frac{1}{\alpha }-\coth\left(\alpha \right)\;\mathrm{and}\;\left\langle \cos^{3}\;\gamma \right\rangle =L_{3}\left(\alpha \right)≡\frac{3\left(2+\alpha^{2}\right)-\alpha \left(6+\alpha^{2}\right)\coth\left(\alpha \right)}{\alpha^{3}}$$
A17

Both of these functions are monotonic in α, and can therefore be inverted:

$$\alpha =L_{1}^{-1}\left(\left\langle \cos\;\gamma \right\rangle \right)\;\mathrm{and}\;\alpha =L_{3}^{-1}\left(\left\langle \cos^{3}\;\gamma \right\rangle \right)$$
A18

so that,

$$\left\langle \cos^{3}\;\gamma \right\rangle =L_{3}\left(L_{1}^{-1}\left(\left\langle \cos\;\gamma \right\rangle \right)\right)≡F\left(\left\langle \cos\;\gamma \right\rangle \right)$$
A19

which defines the function F(x) , a monotonic function of *x*. Using this ansatz and defining *z* = ⟨cos γ⟩ we can write eq [Disp-formula eqA17] as,

$$z+F\left(z\right)=2.184\frac{I_{CC}}{I_{SC}}$$
A20

which can be solved for *z*, allowing us to find the average angle between the C≡N groups in the liquid. The experimental value for the ratio $\frac{I_{CC}}{I_{SC}}$ = – 0.15 and eq [Disp-formula eqA20] becomes z+F(z)=−0.337 , which can be numerically solved to yield ⟨ cos γ⟩ = –0.21, which means γ ∼ arccos (⟨cos γ⟩) ∼ 102° ± 2°, where error bars are given based on the experimental noise.

Furthermore, eq [Disp-formula eqA15] also provides experimental boundaries on the *I*_*CC*_/*I*_*SC*_ ratio. Since the maximum value of cos γ = 1, it can be shown that |*I*_*CC*_/*I*_*SC*_| ≤ 0.824, regardless of the type of interactions.
